# TGF-β-mediated epithelial–mesenchymal transition and tumor-promoting effects in CMT64 cells are reflected in the transcriptomic signature of human lung adenocarcinoma

**DOI:** 10.1038/s41598-021-01799-x

**Published:** 2021-11-17

**Authors:** Naoya Miyashita, Takayoshi Enokido, Masafumi Horie, Kensuke Fukuda, Hirokazu Urushiyama, Carina Strell, Hans Brunnström, Patrick Micke, Akira Saito, Takahide Nagase

**Affiliations:** 1 Department of Respiratory Medicine, Graduate School of Medicine, The University of Tokyo, 7-3-1 Hongo, Bunkyo-ku, Tokyo, 113-0033 Japan; 2 Department of Cancer Genome Informatics, Graduate School of Medicine, Osaka University, 2-2 Yamadaoka, Suita, Osaka 565-0871 Japan; 3 Department of Immunology, Genetics and Pathology, Uppsala University, 75185 Uppsala, Sweden; 4 Laboratory Medicine Region Skåne, Department of Clinical Sciences Lund, Pathology, Lund University, 22185 Lund, Sweden

**Keywords:** Cell signalling, Cancer, Cancer models, Lung cancer

## Abstract

Epithelial–mesenchymal transition (EMT) is a cellular process during which epithelial cells acquire mesenchymal phenotypes. Cancer cells undergo EMT to acquire malignant features and TGF-β is a key regulator of EMT. Here, we demonstrate for the first time that TGF-β could elicit EMT in a mouse lung adenocarcinoma cell line. TGF-β signaling activation led to cell morphological changes corresponding to EMT and enhanced the expression of mesenchymal markers and EMT-associated transcription factors in CMT64 lung cancer cells. RNA-sequencing analyses revealed that TGF-β increases expression of Tead transcription factors and an array of Tead2 target genes. TGF-β stimulation also resulted in alternative splicing of several genes including Cd44, tight junction protein 1 (Tjp1), and Cortactin (Cttn). In parallel with EMT, TGF-β enhanced cell growth of CMT64 cells and promoted tumor formation in a syngeneic transplantation model. Of clinical importance, the expression of TGF-β-induced genes identified in CMT64 cells correlated with EMT gene signatures in human lung adenocarcinoma tissue samples. Furthermore, TGF-β-induced gene enrichment was related to poor prognosis, underscoring the tumor-promoting role of TGF-β signaling in lung adenocarcinoma. Our cellular and syngeneic transplantation model would provide a simple and useful experimental tool to study the significance of TGF-β signaling and EMT.

## Introduction

Lung cancer is the leading cause of cancer-related mortality worldwide and adenocarcinoma is the most frequent histological subtype. Recent advances in comprehensive cancer genome profiling have revealed distinct molecular aberrations in lung adenocarcinoma^1^. Although a subset of cancer drivers has been exploited for therapeutic intervention, cancer cells generally acquire drug resistance to molecular targeted therapies^2^. In recent years, immune checkpoint inhibitors have contributed to the improvement of disease control and patient survival in lung cancer. Nevertheless, durable clinical responses of immunotherapy are observed in a minority of cases^3^.

For preclinical studies to test drug efficacy, an appropriate animal model is necessary. To date, most studies have utilized xenograft models where human cancer cells are transplanted into immunodeficient mice. This implies a major limitation, considering the importance of tumor immune response. The analysis of tumorigenesis in immunocompetent mice is more relevant in respect to lung cancer pathogenesis and its clinical impact. Transplantation of Lewis lung cancer cells in syngeneic immunocompetent mice has frequently been used in previous studies^4^. However, this cell type is highly aggressive and lacks epithelial features, questioning its relevance as an experimental model for lung cancer. Consequently, genetically engineered mice have been exploited to study molecular mechanisms of lung cancer pathogenesis. They currently present the best option to study host-tumor interactions, although such models are costly and time-consuming in general^5^.

Epithelial–mesenchymal transition (EMT) is a cellular process during which epithelial cells acquire mesenchymal phenotypes. EMT is associated with malignant features and drug resistance of lung cancer cells^6^. In the past decade, A549 human lung adenocarcinoma cell line was most frequently used to study EMT. These cell culture studies have demonstrated that EMT endows cells with invasive phenotypes and molecular processes related to EMT have been characterized in detail^7^. Although, based on the limitation of this model, the pathological relevance and clinical significance of EMT in lung cancer remains inconclusive.

TGF-β is a central regulator of EMT and is known to induce EMT-associated transcription factors such as SNAI1, SNAI2, ZEB1, ZEB2, and HMGA2^8^. We have previously demonstrated that TGF-β coordinately upregulates a set of genes related to cell adhesion, cytoskeletal rearrangement, and extracellular matrix (ECM) remodeling during the process of EMT^7^. Recent studies further revealed that TGF-β induces alternative splicing of several genes through expression changes in RNA binding proteins such as epithelial splicing regulatory proteins 1 and 2 (ESRP1 and ESRP2)^9^.

The aim of this study was to characterize and cross-validate TGF-β-mediated EMT in CMT64 mouse lung adenocarcinoma cell line and its tumorigenesis in a syngeneic transplantation model.

## Results

### TGF-β elicits EMT in CMT64 mouse lung adenocarcinoma cells

CMT 64 cells were established from an alveologenic lung carcinoma in a C57BL/lcrf mouse, which have a Kras-activating mutation and wild-type p53 gene^10^. We have recently demonstrated that orthotopically injected CMT64 cells form lung tumors with mediastinal lymphadenopathy in C57BL/6 mice^11^. To further characterize this cell line, we tested whether CMT64 cells could undergo EMT by TGF-β.

**Figure 1:**
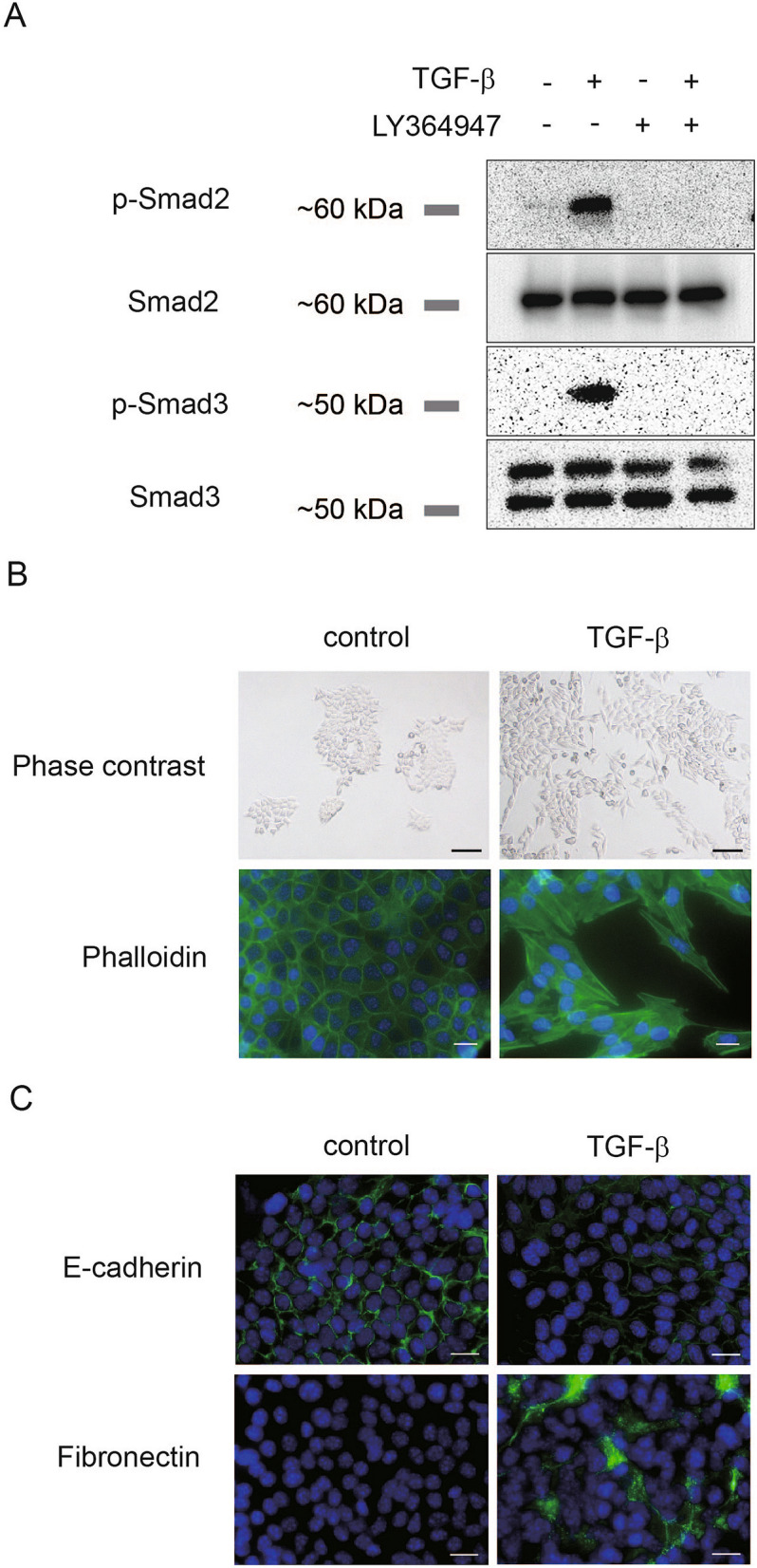
TGF-β induces EMT in CMT64 lung adenocarcinoma cells. (**A**) CMT64 cells were pretreated with 3 μM of LY364947, a TGF-β type I kinase inhibitor, or control DMSO, and were further stimulated with 5 ng/ml of TGF-β for 1 h. LY364947 or DMSO were added 1 h before TGF-β treatment. Immunoblotting was performed for phosphorylated Smad2 (p-Smad2; C-terminal region, Ser 465/467), total Smad2, phosphorylated Smad3 (p-Smad3; C-terminal region, Ser 423/425), and total Smad3. (**B**) CMT64 cells were cultured with or without 5 ng/ml TGF-β for 72 h. Upper: Cells were analyzed by phase-contrast microscopy (bar: 100 µm). Lower: Actin reorganization was visualized by phalloidin staining (bar: 20 µm). Green: phalloidin. Blue: DAPI (nuclei). (**C**) CMT64 cells were incubated with or without 5 ng/ml of TGF-β for 72 h. Immunocytochemistry for E-cadherin or fibronectin (green) was performed. Blue: DAPI (nuclei).

We first investigated cellular responsiveness to TGF-β. It is known that TGF-β binds to type II and type I serine/threonine kinase receptors, and upon ligand binding, Smad2 and Smad3 are phosphorylated by TGF-β type I receptor^12^. In CMT64 cells, Smad2 and Smad3 were clearly phosphorylated upon TGF-β treatment and this effect was abrogated by LY364947, a TGF-β type I kinase inhibitor (Fig. 1A). These results indicated that the intracellular Smad pathway is activated in CMT64 cells in response to TGF-β.

Cultured CMT64 cells formed adherent cell colonies that showed a cobblestone appearance (Fig. 1B, upper left). In contrast to untreated control cells, TGF-β triggered a drastic morphological change to an elongated appearance and enhanced cell dissociation and scattering (Fig. 1B, upper right). Phalloidin staining revealed cell cytoskeletal changes evoked by TGF-β (Fig. 1B, lower).

EMT is accompanied by expression changes in cell adhesion molecules. Immunofluorescence showed attenuated staining for E-cadherin and stronger positivity for fibronectin in CMT64 cells treated with TGF-β (Fig. 1C). These observations indicated that TGF-β elicits EMT in CMT64 cell as determined by cell morphology and EMT markers.

### Transcriptome analysis of CMT64 cells treated with TGF-β and/or TNF-α

**Figure 2:**
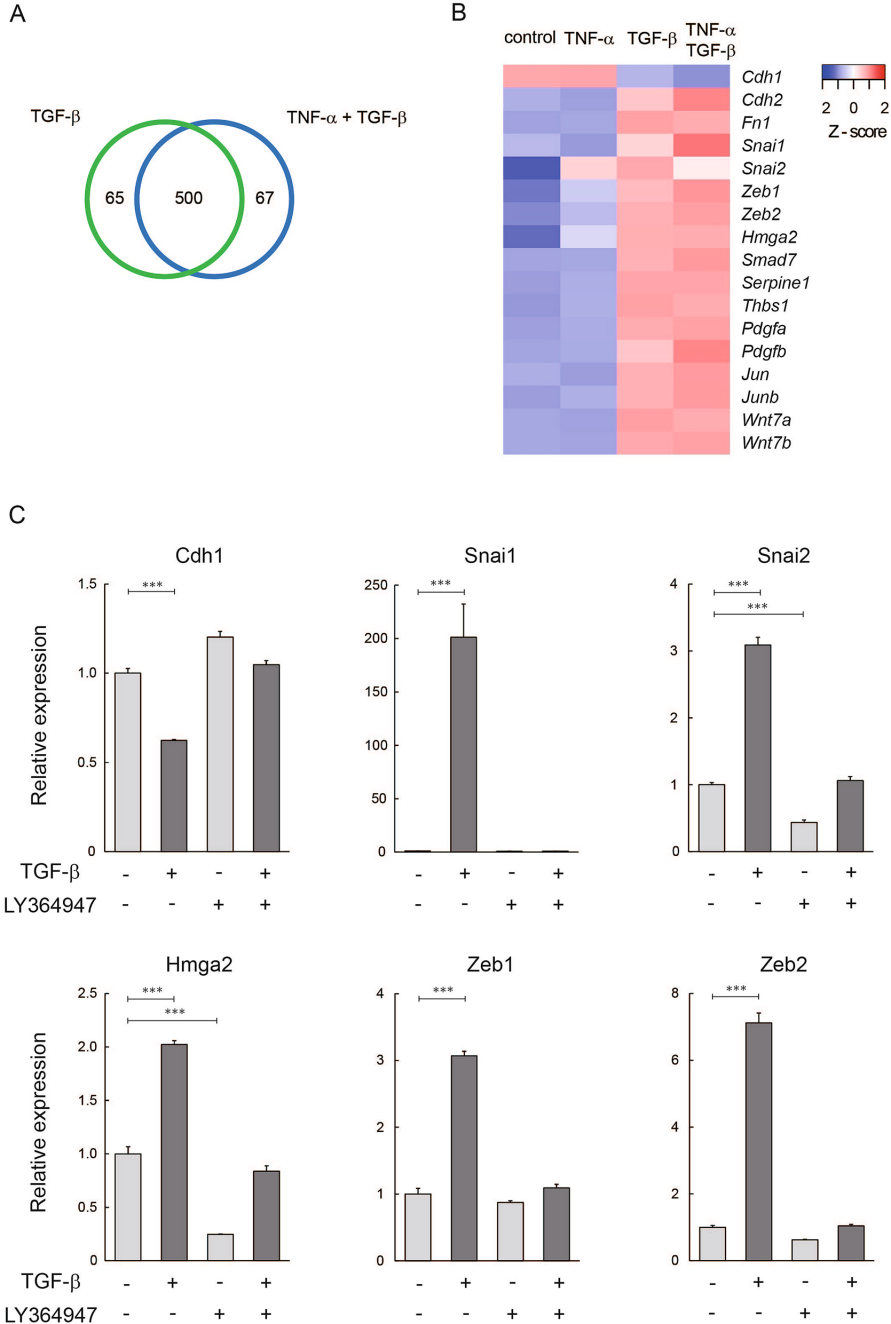
Gene expression profiling following TGF-β and/or TNF-α stimulation. (**A**) Venn diagram illustrating overlaps between genes upregulated by TGF-β alone and TGF-β/TNF-α costimulation in CMT64 cells. The threshold was set as fold change > 2 and RPKM > 2 in the group treated with TGF-β alone or both TGF-β and TNF-α. Numbers of identified genes are indicated. (**B**) Heatmap indicates relative expression levels of EMT markers (Cdh1, Cdh2, and Fn1), EMT-associated transcription factors (Snai1, Snai2, Zeb1, Zeb2, and Hmga2), and TGF-β target genes (Smad7, Serpine1, Thbs1, Pdgfa, Pdgfb, Jun, Junb, Wnt7a, and Wnt7b). CMT64 cells were treated with 5 ng/ml of TGF-β and/or 10 ng/ml TNF-α for 24 h. For the indicated genes, z-scores were calculated from RPKM values obtained by RNA-seq analysis. (**C**) Quantitative RT-PCR was performed for Cdh1 and EMT-associated transcription factors (Snai1, Snai2, Zeb1, Zeb2, and Hmga2) in CMT64 cells treated with 5 ng/ml of TGF-β for 24 h in the presence or absence of 3 μM of LY364947. Expression levels were normalized to that of Gapdh. Experiments were performed in triplicate. Error bars: SE.

We have previously demonstrated that TNF-α, a proinflammatory cytokine, enhances TGF-β-mediated EMT in A549 human lung cancer cells^13^. We further identified subsets of genes differentially or cooperatively regulated by TGF-β and/or TNF-α by microarray analyses^7^. In this study, we carried out RNA-seq analyses and explored gene expression changes in CMT64 cells following TGF-β and/or TNF-α treatment (Supplementary Table S1). Using the threshold of fold change > 2 and RPKM > 2 in TGF-β-treated cells, 565 genes were found to be upregulated by TGF-β. Most of them (500 genes) were also induced by costimulation of TGF-β and TNF-α (Fig. 2A). This result indicated that the TGF-β-mediated regulation of gene expression was coherent even after the proinflammatory stimulation.

To infer functional categories related to TGF-β-induced genes, we performed gene ontology analysis using the Enrichr webtool^14^. The molecular function terms included “transforming growth factor beta binding” and “integrin binding”, confirming the validity of the analyzed gene set (Supplementary Fig. S1).

The predicted biological processes included “regulation of cell migration”, “extracellular matrix organization”, and “(positive) regulation of cell proliferation” (Supplementary Fig. S1).

RNA-seq results confirmed that Cdh1 encoding E-cadherin was downregulated while Cdh2 encoding N-cadherin and fibronectin (Fn1) were upregulated by TGF-β (Fig. 2B). We also noted increased expression levels of EMT-associated transcription factors (Snai1, Snai2, Zeb1, Zeb2, and Hmga2) and previously characterized TGF-β target genes (Smad7, Serpine1, Thbs1, Pdgfa, Pdgfb, Jun, Junb, Wnt7a, and Wnt7b) in TGF-β-treated CMT64 cells, and these results were in accordance with our previous observation in A549 human lung cancer cells^7,15^ (Fig. 2B).

We next validated TGF-β-mediated expression changes in Cdh1 and EMT-associated transcription factors. Quantitative RT-PCR analyses revealed that Cdh1 was downregulated whereas Snai1, Snai2, Zeb1, Zeb2, and Hmga2 were induced by TGF-β (Fig. 2C). Importantly, these effects were abrogated by TGF-β signaling inhibition by LY364947 pretreatment (Fig. 2C). TGF-β-mediated upregulation of soluble factors (Pdgfa, Pdgfb, Wnt7a, and Wnt7b) was also validated by quantitative RT-PCR (Supplementary Fig. S2). These results suggested that this cell line shows the typical EMT-related gene expression profile in response to TGF-β treatment.

### TGF-β-mediated EMT is accompanied by enhanced activity of Tead transcription factors

**Figure 3:**
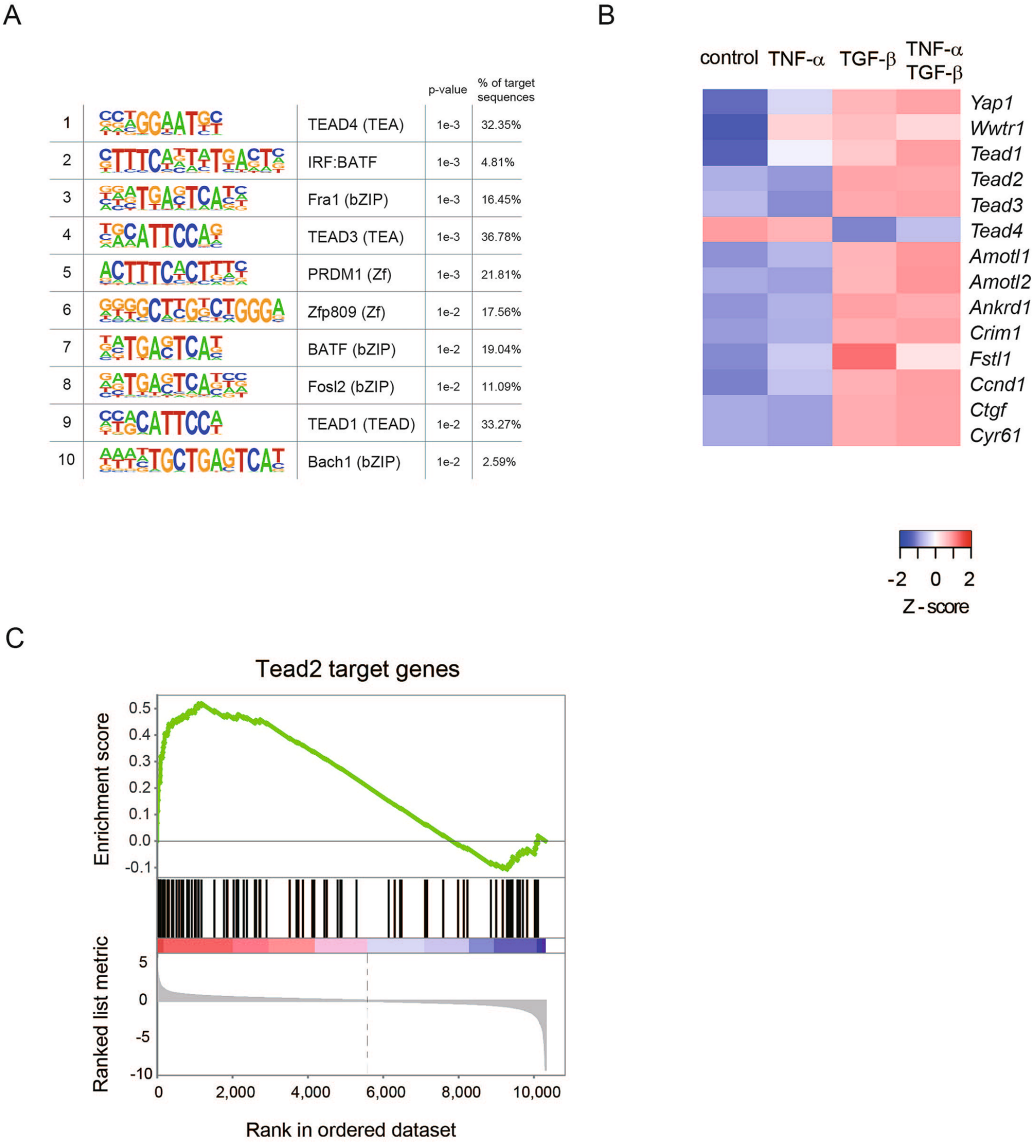
TGF-β-mediated EMT is accompanied by enhanced activity of Tead transcription factors. (**A**) Three known recognition sequences for TEAD transcription factors were among top 10 enriched motifs at the promoters of genes induced by TGF-β. Genes without changes upon TGF-β treatment were used as backgrounds. Percentage of genes that have the indicated motif is shown. (**B**) Heatmap indicates relative expression levels of Yap/Taz signaling components (Yap1, Wwtr1, Tead1, Tead2, Tead3, and Tead4) and Yap/Taz target genes (Amotl1, Amotl2, Crim1, Fstl1, Ccnd1, Ctgf, and Cyr61). CMT64 cells were treated with 5 ng/ml of TGF-β and/or 10 ng/ml TNF-α for 24 h. For the indicated genes, z-scores were calculated from RPKM values obtained by RNA-seq analysis. (**C**) Gene set enrichment analysis was performed to examine the enrichment of Tead2 target gene signature in TGF-β-induced genes in CMT64 cells.

To identify common regulatory elements of TGF-β-induced genes identified in the RNA-seq data, we performed a motif analysis. Strikingly, recognition sequences for TEAD family transcription factors were enriched in the promoter regions of TGF-β-induced genes and were ranked within the top 10 as determined by p-value sorting (Fig. 3A). The RNA-seq data also indicated that transcript levels of Tead transcription factors increased by TGF-β stimulation. Among them, the expression level of Tead2 was prominent in CMT64 cells and was clearly upregulated by TGF-β (Supplementary Fig. S3).

Tead transcription factors are known to interact with transcriptional co-activators, Yap (encoded by Yap1) and Taz (encoded by Wwtr1), which are reportedly implicated in the regulation of EMT^16^. Interestingly, transcript levels of Yap1 and Wwtr1 also tended to increase by TGF-β stimulation (Fig. 3B). In accordance, previously characterized Yap/Taz target genes (Amotl1, Amotl2, Ankrd1, Crim1, Fstl1, Ccnd1, Ctgf, and Cyr61)^17^ were upregulated by TGF-β (Fig. 3B). A recent report has shown that TGF-β-mediated Tead2 upregulation could contribute to EMT in mouse breast cancer cells^18^. Gene set enrichment analysis indicated that previously identified Tead2 target genes^18^ were enriched among TGF-β-induced genes in CMT64 cells (Fig. 3C).

### Differential splicing is induced by TGF-β in CMT64 cells

To gain deeper insights into the transcriptional changes induced by TGF-β, we aimed to identify alternative splicing in CMT64 cells using the RNA-seq data. Comprehensive profiling of alternative transcripts was performed using the rMATS protocol^19^. Among the profiled transcripts, we noted that TGF-β elicits alternative splicing of several genes involved in ECM-cell interaction, cell junction, and cytoskeletal organization (Supplementary Table S2).

**Figure 4:**
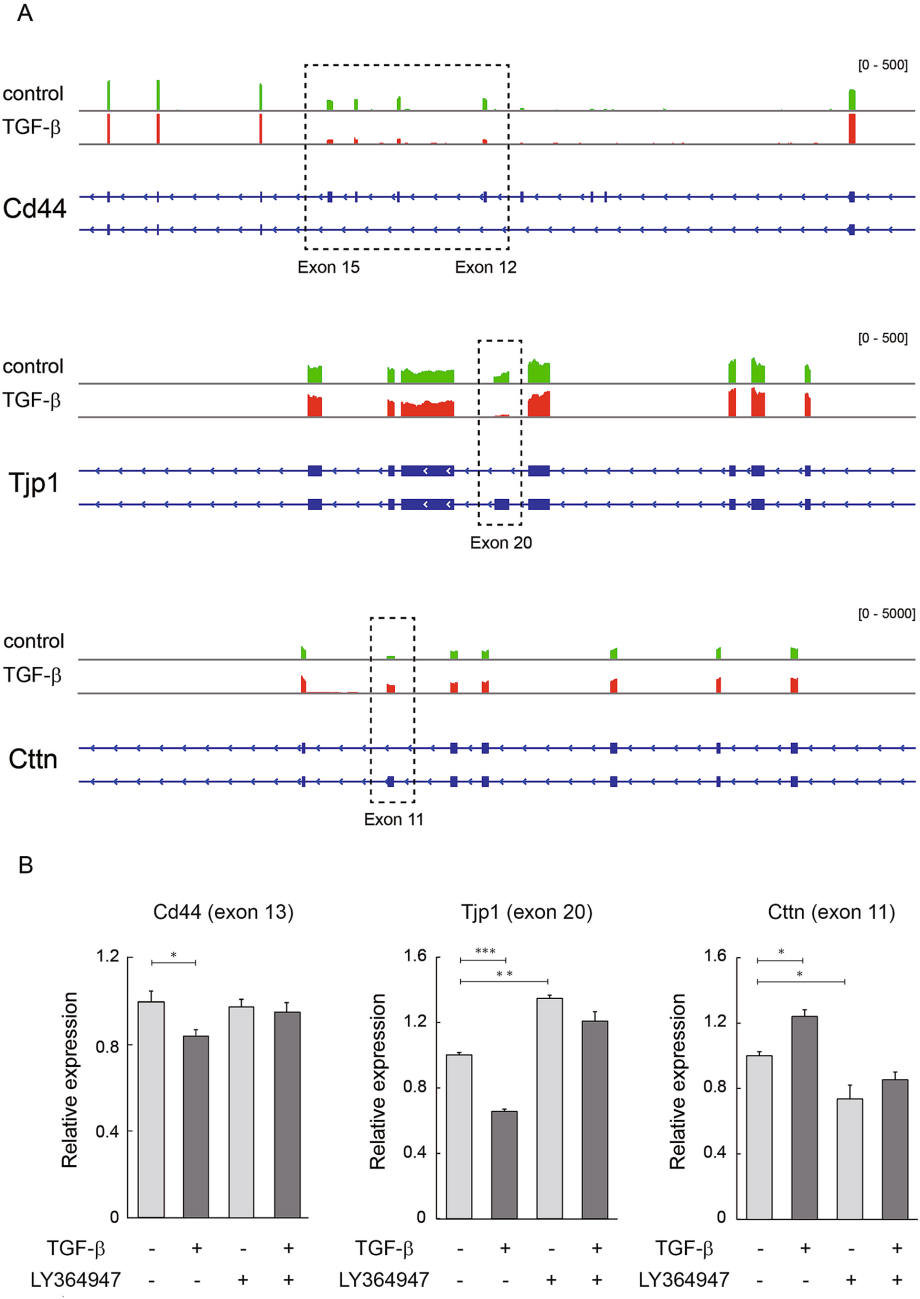
Differential splicing is induced by TGF-β in CMT64 cells. (**A**) Alternative transcripts for genes involved in ECM-cell interactions (Cd44), cell junction (Tjp1), and cytoskeletal organization (Cttn). Dotted square indicates the exons of alternative splicing by TGF-β treatment. Expression levels determined by RNA-seq in CMT64 cells cultured with or without 5 ng/ml of TGF-β for 24 h were shown by Integrative Genomics Viewer. (**B**) Quantitative RT-PCR was performed for Cd44 exon 13, Tjp1 exon 20, and Cttn exon 11 in CMT64 cells treated with 5 ng/ml of TGF-β for 24 h in the presence or absence of 3 μM of LY364947. LY364947 or DMSO was added 1 h before TGF-β treatment. Expression levels were normalized to that of Gapdh. Error bars: SE.

Epithelial isoforms of the cell surface protein Cd44 (Cd44v) contain variant exons with different combinations whereas the mesenchymal (standard) isoform (Cd44s) is devoid of all variant exons. TGF-β elicited isoform switching of Cd44 mRNA from Cd44v7-10 to CD44s and the expression levels of exon 12 to exon 15 were reduced by TGF-β (Fig. 4A). TGF-β-mediated downregulation of Cd44 exon 13 was confirmed by quantitative RT-PCR, indicating the epithelial–mesenchymal switch of Cd44 (Fig. 4B).

Similarly, TGF-β treatment led to an increase in variant transcripts of the tight junction protein 1 (Tjp1, also known as ZO-1) devoid of exon 20 in CMT64 cells (Fig. 4A). A previous report demonstrated that exon 20 skipping of Tjp1 enhances cytoskeletal alterations during EMT in A549 cells^20^. Quantitative RT-PCR confirmed that the expression level of Tjp1 exon 20 decreased by TGF-β stimulation (Fig. 4B).

Cortactin (Cttn) acts as an actin assembly protein and regulates cytoskeletal dynamics. It has been reported that cells expressing Cttn splicing variants that lack exon 11 show reduced cell migration^21^. Consistent with the observation that TGF-β promoted cell scattering (Fig. 1B), the expression levels of Cttn exon 11 increased by TGF-β stimulation in CMT64 cells and this effect was confirmed by quantitative RT-PCR (Fig. 4B).

### TGF-β promotes cell growth and tumorigenesis of CMT64 cells

TGF-β generally exerts an inhibitory effect on epithelial cell proliferation^22^ and conceptually TGF-β acts as a tumor suppressor in the early stage of tumorigenesis. Conversely, in advanced stages, TGF-β promotes cancer progression by both cell-autonomous mechanisms and modulations of the tumor microenvironment^23^. Although molecular mechanisms of EMT have been widely studied, it is poorly understood whether and how TGF-β-mediated EMT is connected to cancer cell proliferation.

**Figure 5:**
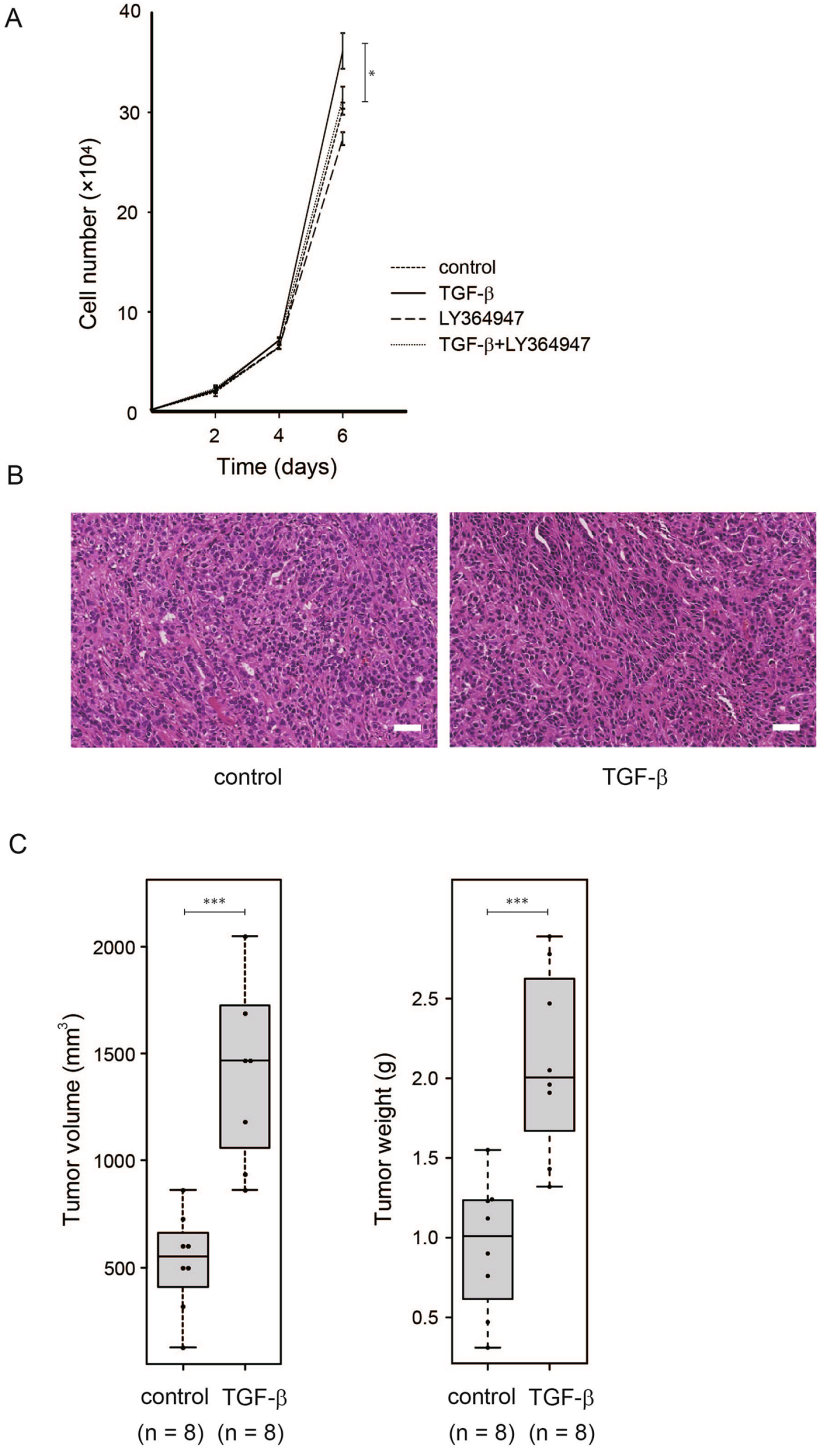
TGF-β enhances cell growth and tumorigenesis of CMT64 cells. (**A**) Cell count of CMT64 cells on days 2, 4, and 6 after 5 ng/ml of TGF-β stimulation are shown. (**B**) Hematoxylin & eosin staining image of tumors derived from CMT64 cells. CMT64 cells treated with or without 5 ng/ml of TGF-β for 96 h were injected subcutaneously into C57BL/6 mice. CMT64 cells were exposed to TGF-β only at the pre-transplant stage. The tumor was harvested after 26 d after subcutaneous injection of 1 × 10^7^ CMT64 cells. Bar: 50 μm. (**C**) CMT64 cells treated with or without 5 ng/ml of TGF-β for 96 h were injected subcutaneously into C57BL/6 mice. Tumor volume and tumor weight were measured after 17 days. Eight mice were used in both groups. P < 0.05 for each measurement; Student’s t-test.

In CMT64 cells, TGF-β slightly enhanced cell growth and this effect was inhibited by LY364947 (Fig. 5A). To test whether TGF-β could influence tumor formation in syngeneic mice, CMT64 cells treated with or without TGF-β were subcutaneously transplanted into C57BL/6 mice. Tumors derived from untreated CMT64 cells showed histological appearances of differentiated adenocarcinoma and we did not find an apparent morphological difference between the tumors derived from untreated and TGF-β-treated cells (Fig. 5B).

In accordance with the results of cell culture experiments (Fig. 5A), TGF-β-treated CMT64 cells formed statistically larger tumors in size and weight compared to untreated cells (Fig. 5C). Because CMT64 cells were treated with TGF-β prior to transplantation, TGF-β-induced EMT might lead to a growth advantage at the initial stage of tumorigenesis after subcutaneous injection into mice.

### Clinical relevance of TGF-β-mediated EMT in lung cancer

By analyzing the RNA-seq data of CMT64 cells treated with TGF-β, we identified 565 genes upregulated by TGF-β using the threshold of fold change > 2 and RPKM > 2 in TGF-β-treated cells. On the other hand, 260 genes were found to be downregulated by TGF-β using the threshold of fold change < 0.25 and RPKM > 1 in untreated cells. Among them, we could identify 520 upregulated and 228 downregulated human orthologs (Supplementary Table S3). We calculated TGF-β-induced gene signature score (hereafter referred to as TGF-β score) by subtracting the average expression level of downregulated genes from that of upregulated genes. We did not find any correlations between TGF-β scores and evaluated clinical parameters (sex, age, smoking status, stage at diagnosis, and performance status) (Supplementary Table S4).

To further explore clinical relevance of TGF-β score derived from TGF-β-treated CMT64 cells, we compared it with EMT and ECM scores based on EMT and ECM gene signatures reported by Mak et al. and Chakravarthy et al., respectively (Supplementary Table S3)^24,25^. Seven of 30 ECM-upregulated genes and 10 of 52 mesenchymal markers were found to be upregulated by TGF-β in CMT64 cells. On the other hand, one of 28-ECM downregulated genes and two of 25 epithelial markers were downregulated by TGF-β in CMT64 cells (Supplementary Fig. S4).

**Figure 6:**
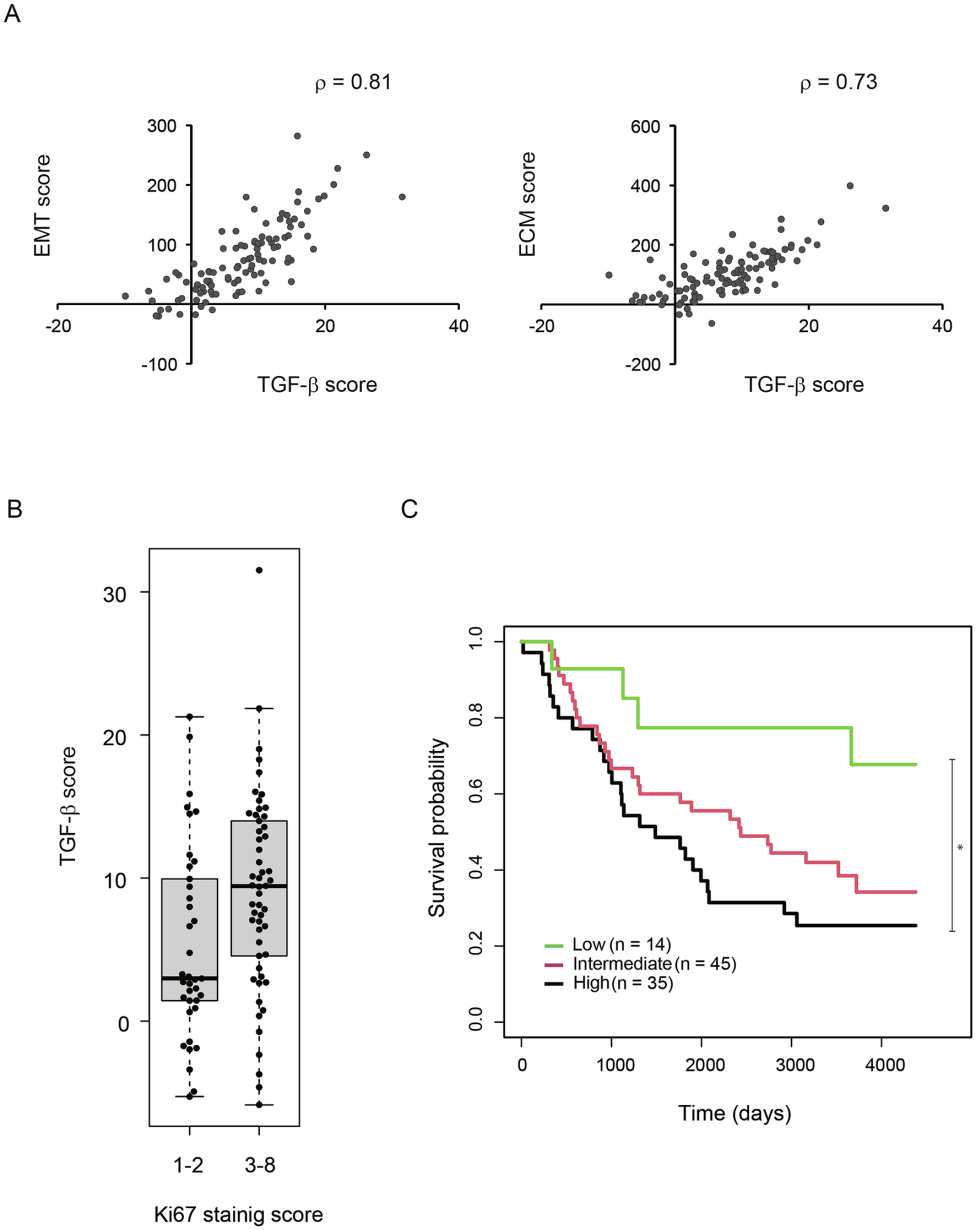
Clinical relevance of TGF-β-mediated gene expression changes. (**A**) Correlation between TGF-β-induced gene signature scores and those of EMT or ECM in lung adenocarcinoma tissue samples derived from the GSE81089 dataset. (**B**) Box plot of TGF-β scores were compared between two subgroups of staining scores for Ki67. (**C**) Kaplan–Meier analysis of lung adenocarcinoma cases in the GSE81089 dataset by stratification of TGF-β-induced gene signature scores.

We analyzed the RNA-seq data of lung adenocarcinoma tissue samples obtained at Uppsala University Hospital (GSE81089) and investigated relationships among these gene signatures. TGF-β, EMT, and ECM scores were calculated based on the expression levels of signature genes (Supplementary Table S3). Strikingly, TGF-β scores strongly correlated with those of EMT or ECM in the analyzed lung adenocarcinoma tissue samples (Fig. 6A).

As TGF-β treated CMT64 cells formed larger tumors in mice (Fig. 5C), we investigated the relationship between TGF-β scores and tumor proliferation in human lung adenocarcinoma tissue samples. Immunohistochemistry for Ki67 was performed and notably, tumors with higher Ki67 staining scores showed higher TGF-β scores. This observation suggested that TGF-β signaling activation is associated with higher proliferation levels of lung adenocarcinomas (Fig. 6B).

Finally, we explored the association between lung adenocarcinoma patient prognosis and TGF-β scores: high: > 10 (n = 35), intermediate: 0–10 (n = 45), and low: < 0 (n = 14). Of clinical importance, high TGF-β scores were associated with shorter survival time (Supplementary Fig. S5) and were predictive of poor prognosis in lung adenocarcinoma patients (Fig. 6C). These observations suggested that TGF-β-mediated EMT defines prognostic subgroups of lung adenocarcinoma patients, and therefore suggest that the CMT64 model is of translational relevance.

## Discussion

In this study, we revealed that TGF-β elicits EMT in CMT64 mouse lung adenocarcinoma cells and TGF-β-treated CMT64 cells form larger tumors in vivo. This cellular model recapitulated molecular mechanisms associated with TGF-β signaling and the transcriptomic data provided extended information about genes involved in TGF-β-mediated EMT. The TGF-β-induced gene profile in CMT64 cells was independently mirrored by EMT and ECM gene signatures, and was of prognostic impact in human lung adenocarcinoma patients, which substantiated the validity of this model. To our knowledge, this is the first demonstration of EMT in mouse lung cancer cells, providing additional evidence of the relevance of TGF-β signaling in lung tumorigenesis.

Recently, EMT international association proposed definitions for research on EMT^26^. It is thought that EMT encompasses a wide range of activation status and most cancer cells already exist in an intermediate state in the spectrum between epithelial and mesenchymal differentiation. CMT64 cells formed adherent cell colonies with high E-cadherin expression, suggesting that this cell type resides closely to an epithelial state in the EMT spectrum.

As TGF-β generally exerts cytostatic effects on epithelial cells and even various human lung cancer cells^27^, our observation that TGF-β could stimulate cell growth and tumor formation of CMT64 cells might provide a clue to understanding molecular mechanisms how TGF-β has the tumor-promoting role in the later stage of cancer progression.

By analyzing the RNA-seq data, we also comprehensively profiled splicing events in CMT64 cells treated with TGF-β. Noteworthy, we identified alternative transcripts possibly involved in ECM-cell interactions (Cd44), cell junction (Tjp1), and cytoskeletal organization (Cttn) as molecular candidates for morphological changes during EMT. Importantly, our list of TGF-β-mediated alternative transcripts includes additional information to study post-transcriptional regulation of EMT-related genes.

Interestingly, TGF-β stimulated the expression of secreted proteins, including Pdgfa, Pdgfb, Wnt7a, and Wnt7b, all of which could activate surrounding mesenchymal cells, indicating that EMT is occurring in conjunction with alterations in the tumor microenvironment and involves reciprocal interactions between tumor and stromal cells. This finding once again underlines the need to study EMT in a multi-cellular context.

Xenotransplantation of human lung cancer cells into immunodeficient mice insufficiently provides the information about tumor-stromal interactions and does not resemble the complex immune environment in human cancer tissues. Importantly, our cellular model can be applied as a syngeneic transplantation assay, which would provide a simple and useful experimental tool to study the pathological significance of TGF-β signaling and EMT. Syngeneic mouse models are of fundamental importance for preclinical drug-testing in an immunocompetent environment and are clearly warranted to analyze the multifaceted action of TGF-β. Several TGF-β inhibitors, targeting the ligands or the receptors are now under development or in clinical trials in different cancer types^28^. The CMT64 syngeneic model allows proof of concept studies to evaluate the efficacy, mechanism of action, and the pharmacodynamics of such TGF-β inhibitors for the treatment of lung adenocarcinoma.

However, lung adenocarcinomas are notoriously heterogeneous in terms of histological appearances and genomic alterations and thus experimental results in our model based on a single cell line should be interpreted with caution. It should also be noted that the clinical relevance of TGF-β-induced EMT shown in our study is suggested based on transcriptomic findings of RNA-seq data. Further studies are needed to confirm that the expression changes are translated to protein levels. Furthermore, EMT can be stimulated by a variety of factors, such as Ras signals, fibroblast growth factor (FGF) or mechanical stress^16,29^. Therefore, it is likely that TGF-β stimulation reflects a part of the complete spectrum of EMT phenotype.

In summary, CMT64 mouse model presents a reliable experimental platform to study molecular mechanisms of EMT. Our study recapitulates known molecular mechanisms but also provides novel insights to understand the pathological role of TGF-β signaling in lung cancer tumorigenesis. To date, the clinical role of TGF-β in human lung cancer is still fragmentarily understood. We hope that our model gives an opportunity to evaluate new strategies to target the tumor-promoting effects of TGF-β.

## Materials and methods

### Cell culture and reagents

CMT64 cells (ECACC Cat# 86082105, RRID:CVCL_4146) were obtained from ECACC (Salisbury, UK). All experiments were performed with mycoplasma-free cells. TGF-β1 and TNF-α were purchased from Sigma-Aldrich (St. Louis, MO) and R&D Systems (Minneapolis, MN), and were used at the concentrations of 5 ng/ml and 10 ng/ml, respectively. LY-364947 (Cayman Chemical, Ann Arbor, MI) was used at the concentration of 3 µM.

### RNA isolation and RT-PCR

Total RNA was isolated using RNeasy Mini kit (Qiagen, Hilden, Germany). Detailed procedures of RT-PCR were described previously^30^. Gapdh expression levels were used for normalization. PCR primers are shown in Supplementary Table S5.

### Immunoblot analysis

Detailed procedures were described previously^31^. Antibodies for Smad2 (Cell Signaling Technology Cat# 5339, RRID: AB_10626777), phosphorylated Smad2 at Ser 465/467 (#3101), Smad3 (#9513), and phosphorylated Smad3 at Ser 423/425 (#9520) were from Cell Signaling (Beverly, MA). Antibodies for E-cadherin and fibronectin were from BD Pharmingen (Transduction Laboratories, Lexington, KY).

### Immunofluorescence

CMT64 cells were fixed with acetone and methanol, and blocked with 5% normal goat serum in PBS. The cells were incubated with anti-E-cadherin (1:50) or anti-fibronectin (1:100) overnight, and then treated with goat anti-mouse Alexa Fluor 488-conjugated antibody (Life Technologies). Nuclei were stained with DAPI.

### RNA-sequencing (RNA-seq) analysis

RNA-seq reads were analyzed using CLC Genomics workbench software (Qiagen). Reads per kilobase of transcript per million mapped reads (RPKM) data were generated as described previously^32^. Adapter sequences were removed, and read mapping was carried out using the default settings of RNA-Seq analysis package (mismatch cost: 2, insertion cost: 3, deletion cost: 3, length fraction: 0.8, similarity fraction: 0.8, and maximum number of hits for a read set to: 30). Dataset was submitted to the GEO repository (GSE164160).

### Motif analysis

Motif analysis was performed using the HOMER findMotifs.pl program as described previously^33^. For the promoters of TGF-β-induced genes, motifs were defined from − 800 to + 200 nucleotides from the transcription start sites, and genes without changes upon TGF-β treatment were used as backgrounds.

### Alternative splicing

Splice variants and differential splicing in the RNA-seq data were identified using rMATS protocol^19^. Mapped sequence data were visualized using Integrative Genomics Viewer^34^.

### Syngeneic transplantation model

CMT64 cells were resuspended at a cell density of 5 × 10^7^/ml in PBS containing 67 µg/ml of Matrigel (BD Pharmingen). A combination anesthetic was prepared with 0.3 mg/kg of medetomidine, 4.0 mg/kg of midazolam, and 5.0 mg/kg of butorphanol. Mice were anaesthetized by intraperitoneal injection and tumor cells suspended in 200 µl of PBS were injected into the subcutaneous tissue of the right back.

### Public data

RNA-seq data of 108 lung adenocarcinomas treated at Uppsala University Hospital were extracted from the GSE81089 dataset^35^. The 77-gene lung cancer EMT signature^24^ and 58-gene cancer-associated ECM signature^25^ were used for correlation analyses. To calculate EMT scores, the average z-score of 25 epithelial markers was subtracted from that of 52 mesenchymal markers. For the calculation of TGF-β and ECM scores, we performed quantile normalization of RNA-seq data so that each gene has the same mean, median and variance. Using the normalized values, the average expression level of downregulated genes was subtracted from that of upregulated genes (Supplementary Table S3).

### Tissue microarray

Tissue microarrays were constructed based on lung cancer patients operated at Uppsala University Hospital between 2006 and 2010. Detailed procedures were described previously^36^. For antigen retrieval, tissue sections were boiled for 4 min at 125 °C in citrate buffer, pH6 (Lab Vision, Freemont, CA). Primary antibodies for Ki67 (Dako, M7240; MIB-1) were diluted at 1:200 in UltraAb Diluent^®^ (Lab Vision), and the secondary reagent UltraVision LP HRP polymer^®^ (Lab Vision) was used. The stained slides were scanned using an Aperio ScanScope AT2 Slide Scanner (Aperio Technologies, Vista, CA). Protein expression levels were scored by the percentage of stained tumor cells as follows. 0: 0%, 1: ≤ 1%, 2: 2–10%, 3: 11–20%, 4: 21–30%, 5: 31–40%, 6: 41–50%, 7: 51–75%, and 8: > 75%.

### Statistical analysis

Spearman's correlation coefficient (ρ) was calculated for correlation analyses. Differences were examined by Student’s t-test or analysis of variance (ANOVA) with Tukey’s post hoc test with JMP, version 14 (SAS Institute Inc., Cary, NC. RRID:SCR_008567). Fisher’s exact test or Chi-square test was applied to test associations between TGF-β scores and clinical variables. Multivariate Cox regression analysis was performed with inclusion of other prognostic parameters: age, performance status, and stage at diagnosis. Log-rank test with Bonferroni correction was used for survival analysis. *p < 0.05. **p < 0.01. ***p < 0.001.

### Ethical approval

The study using human tissue samples was performed in accordance with the Swedish Biobank Legislation and performed in adherence with the Declaration of Helsinki. The study was approved by the Uppsala Regional Ethical Review Board (2012/532). In this retrospective study, the need for obtaining informed consent for participation was regarded unnecessary according to the Swedish national regulations.

The protocol for animal experimentation was approved by the Ethics Committee for Animal experiments. Animal experiments were performed at the University of Tokyo in strict accordance with the guidelines of the University of Tokyo Animal Experiment Implementation Manual and the Regulations for animal experiments at the University of Tokyo. During the whole course of animal experiments, all efforts were made to minimize sufferings. The authors complied with the ARRIVE guidelines. The study is reported in accordance with ARRIVE guidelines (https://arriveguidelines.org).

## Supplementary Information

Supplementary Figures.

Supplementary Tables.

## Data Availability

All data generated or analyzed during this study are included in this published article.
