# Supplementary material for: TGF-β-mediated epithelial–mesenchymal transition and tumor-promoting effects in CMT64 cells are reflected in the transcriptomic signature of human lung adenocarcinoma
Source: Sci Rep. 2021 Nov 17;11:22380. doi: 10.1038/s41598-021-01799-x (PMC8599691; doi:10.1038/s41598-021-01799-x)
Supplement: Supplementary file 1 — Supplementary Figures. [file 41598_2021_1799_MOESM1_ESM.docx]

**Supplementary Figure**

**TGF-β-mediated epithelial-mesenchymal transition and tumor-promoting effects in CMT64 cells are reflected in the transcriptomic signature of human lung adenocarcinoma**

Naoya Miyashita^1,^ *, Takayoshi Enokido^1^, Masafumi Horie^2^, Kensuke Fukuda^1^, Hirokazu Urushiyama^1^, Carina Strell^3^, Hans Brunnström^4^, Patrick Micke^3^, Akira Saito^1^, Takahide Nagase^1^

^1^ Department of Respiratory Medicine, Graduate School of Medicine, The University of Tokyo, 7-3-1 Hongo, Bunkyo-ku, Tokyo 113-0033, Japan

^2^ Department of Cancer Genome Informatics, Graduate School of Medicine, Osaka University, 2-2 Yamadaoka, Suita, Osaka 565-0871, Japan

^3^ Department of Immunology, Genetics and Pathology, Uppsala University, SE-75185 Uppsala, Sweden

^4^ Lund University, Laboratory Medicine Region Skåne, Department of Clinical Sciences Lund, Pathology, SE-22185 Lund, Sweden.

***Corresponding author:**

Naoya Miyashita

Department of Respiratory Medicine, Graduate School of Medicine,

The University of Tokyo

7-3-1 Hongo, Bunkyo-ku, Tokyo 113-0033, Japan

E-mail: miyashita708-tky@umin.ac.jp

Phone: +81-3-3815-5411

Fax: +81-3-3815-5954


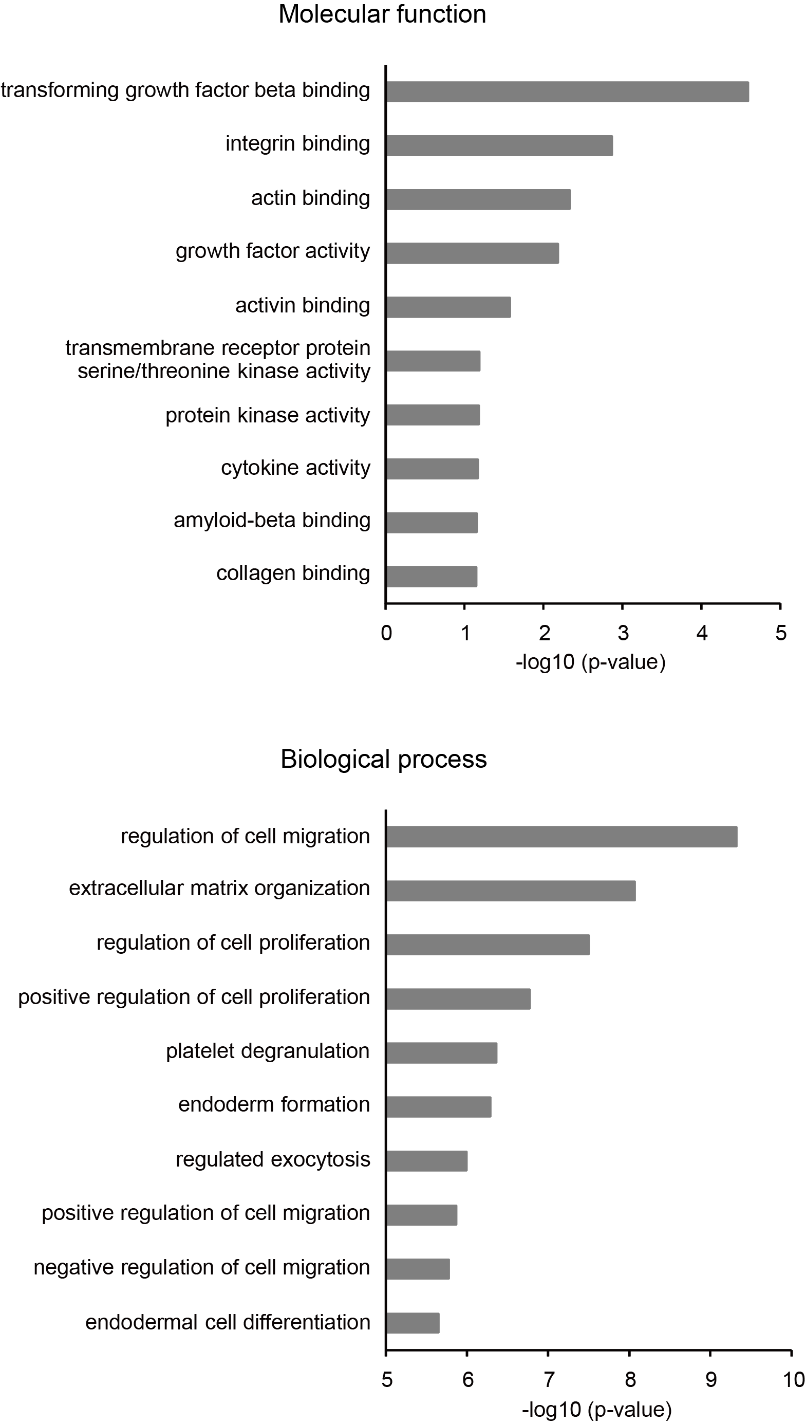


**Supplementary Figure S1**. Gene Ontology analysis of TGF-β-induced genes in CMT64 cells.

Gene ontology analysis of TGF-β-induced genes was performed using the Enrichr webtool. Note that the molecular function terms included “transforming growth factor beta binding” and “integrin binding”, and the predicted biological processes included “regulation of cell migration”, “extracellular matrix organization”, and “regulation of cell proliferation”.


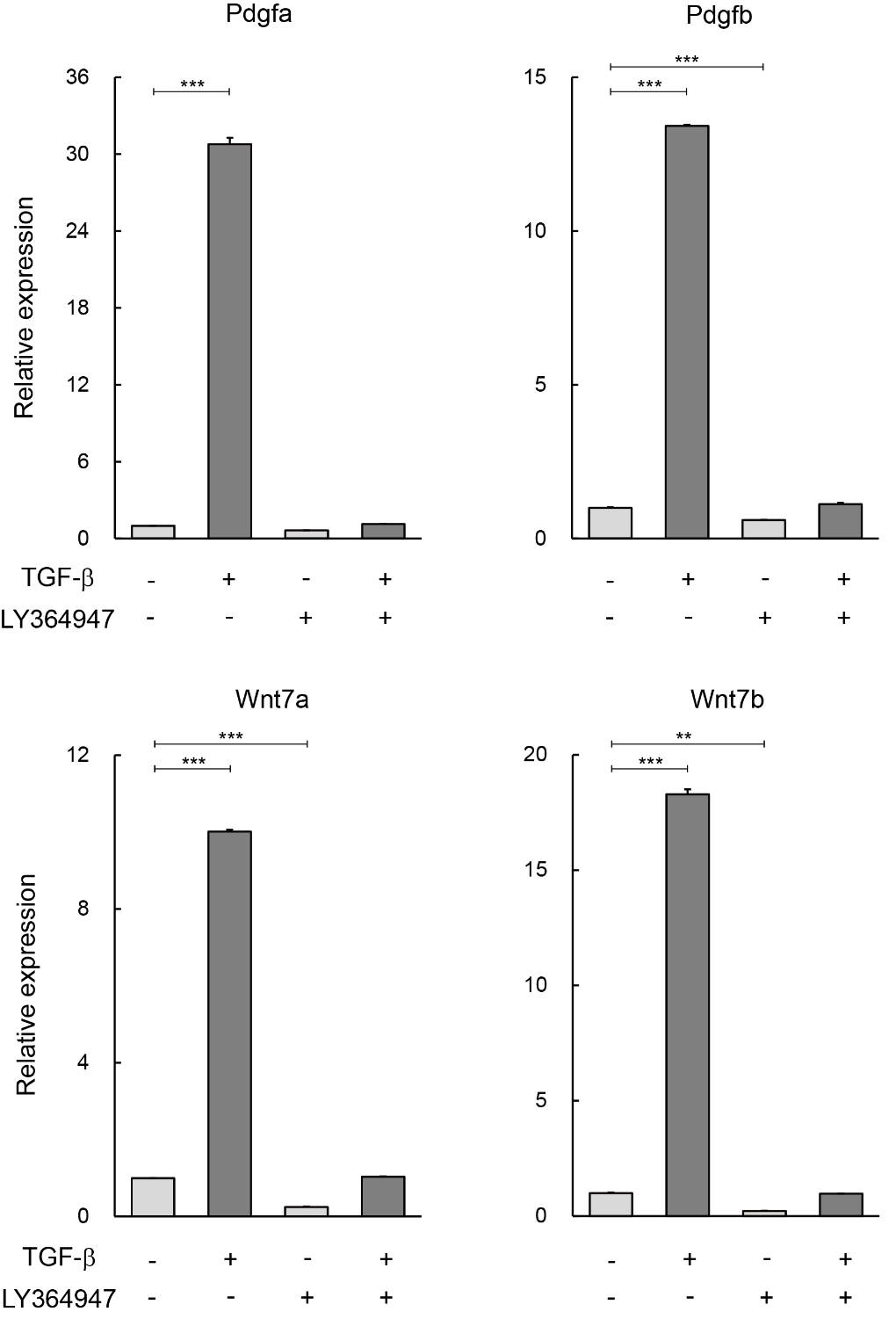


**Supplementary Figure S2**. TGF-β-mediated upregulation of soluble factors.

Quantitative RT-PCR for Pdgfa, Pdgfb, Wnt7a, and Wnt7b in CMT64 cells treated with TGF-β for 24 h in the presence or absence of LY364947. Expression levels were normalized to that of GAPDH. Error bars: SE.


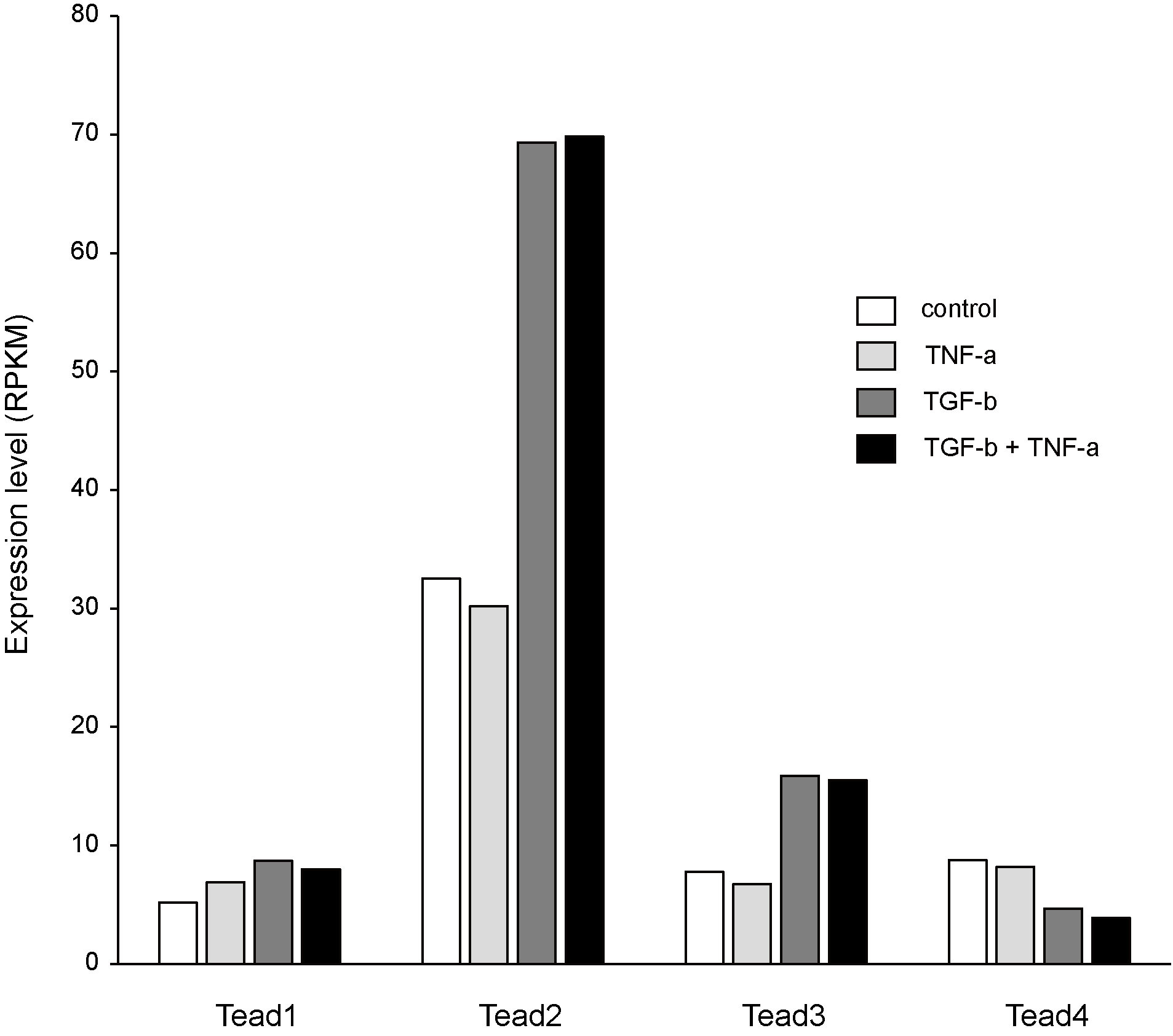


**Supplementary Figure S3**. Transcript levels for Tead transcription factors determined by RNA-seq.

RNA-seq analyses was carried put in CMT64 cells following TGF-β and/or TNF-α treatment. Transcript levels of Tead transcription factors were shown.


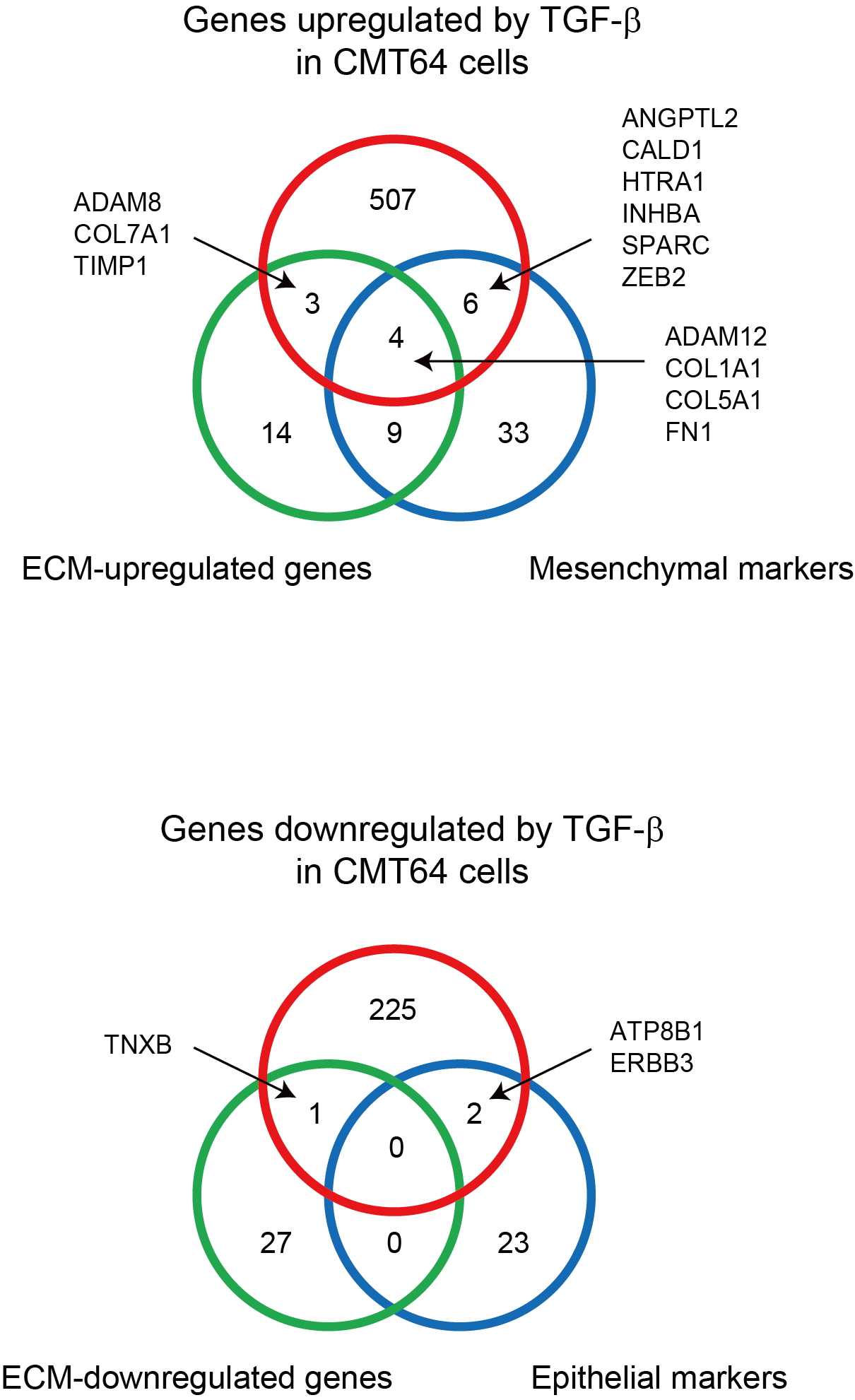


**Supplementary Figure S4**. Genes regulated by TGF-β in CMT64 cells were compared with ECM-regulated genes and mesenchymal/epithelial markers.

Venn diagram showing overlaps of genes up/downregulated by TGF-β in CMT64 cells with ECM-up/downregulated genes (reported by Chakravarthy et al) or mesenchymal/epithelial markers (reported by Mak et al). Numbers of corresponding genes and representative overlapping genes are indicated.


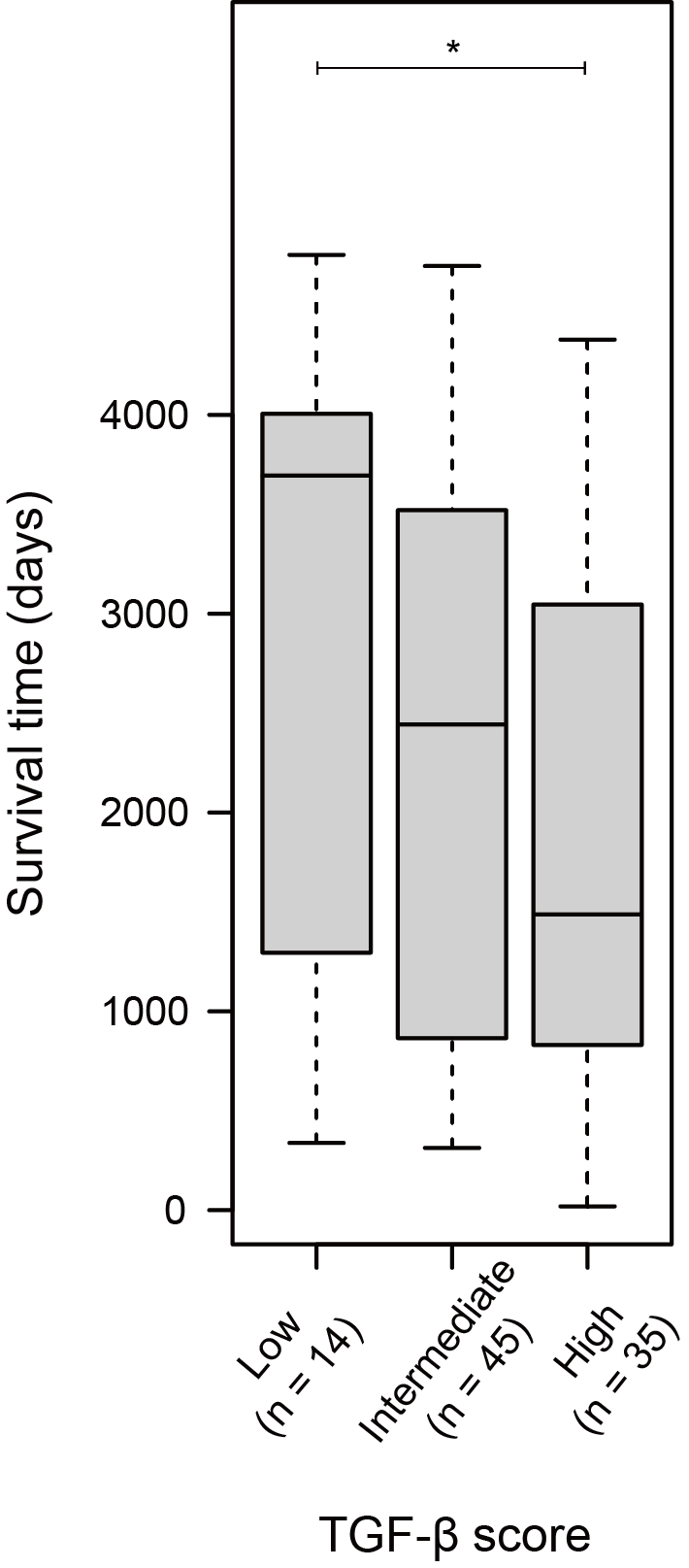


**Supplementary Figure S5**. Association between lung adenocarcinoma patient prognosis and TGF-β scores

Correlation between TGF-β-induced gene signature scores and survival time of lung adenocarcinoma cases in the GSE81089 dataset. Subgroups of TGF-β-induced gene signature scores are classed as high: > 10 (n = 35), intermediate: 0-10 (n = 45), and low: < 0 (n = 14).


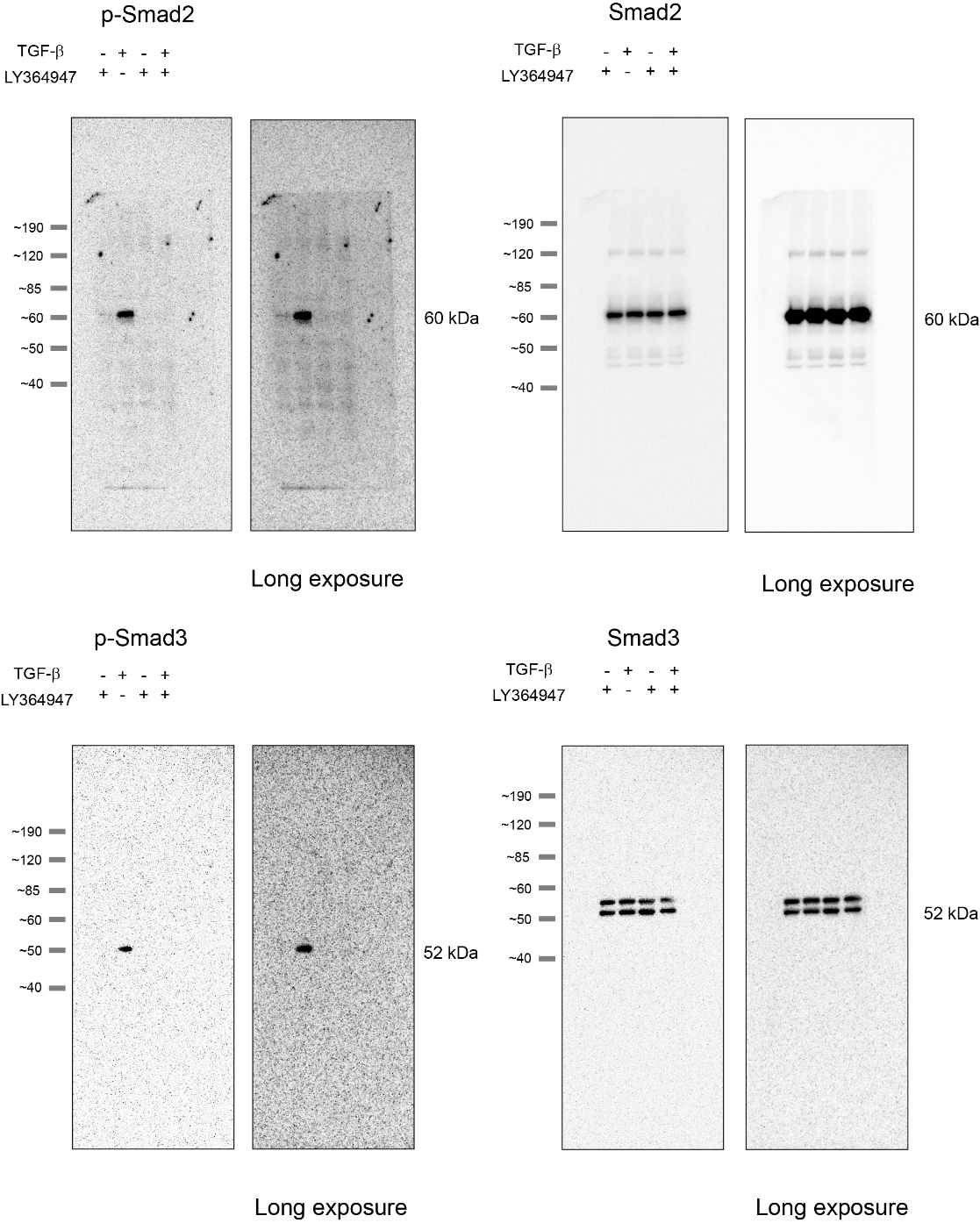


**Supplementary Figure S6**. Full length blots of immunoblot analysis.

Full length blots of p-Smad2, Smad2, p-Smad3, and Smad3 were shown.
